# Cardiovascular Risk Factors: From Consolidated Knowledge to a Call for Action

**DOI:** 10.5935/abc.20150128

**Published:** 2015-10

**Authors:** Guilherme Brasil Grezzana, Lucia Campos Pellanda

**Affiliations:** 1 Fundação Universitária de Cardiologia do Rio Grande do Sul – ICFUC, Porto Alegre, RS – Brazil; 2 Universidade Federal de Ciências da saúde de Porto Alegre – UFCSPA, Porto Alegre, RS – Brazil

**Keywords:** Risk Factors, Cardiovascular Diseases, Prevention, Epidemiologic Studies, Data Interpretation Statistical, Journals Articles

Since the decade of 1950, the most prominent journal in cardiology in Brazil, *Arquivos Brasileiros de Cardiologia (ABC)*, has been indexed in Medline^1^. A total of 7,102 articles have been published since then, on various subjects related to clinical, invasive, surgical cardiology, as well as diagnostic methods.

We performed a systematic review of articles published in ABC during the period from January 2001 to June 2015 containing the MeSH term “cardiovascular risk factors”. Of the 3,087 titles of articles published in the period, 116 articles were identified. The abstracts of these articles were reviewed, and 107 articles, in which assessment of cardiovascular risk factors was the main topic, were included. The sample was composed by 102 original articles, 3 letters and 2 editorials. When specific topics were assessed, “cardiovascular factor or cardiovascular risk” was generally described in 88 articles, 6 articles focused on quality of life and cardiovascular risk factor, 4 articles described epidemiological factors and risk factors, 5 articles specifically explored systemic arterial hypertension and risk factors, 3 were guidelines on risk factors and 1 articles related risk factor with public health. However, when we focused only on isolated risk factors, there has been a clear preponderance of articles involving arterial hypertension (18% between 2010 and 2013) and a trend of increase in the number of articles on diabetes (approximately 10%) published on ABC in the last years.

The average annual number of articles focused on cardiovascular risk factors published on ABC has been consistent in the last 15 years, with a mean of 3.47% of total publications per year, and no significant differences between years (p = 0.195) (Table 1). Considering SciELO database and the number of accesses to the articles selected between January 2014 and June 2015, the 2 most accessed articles were cross-sectional studies on metabolic syndrome and systemic arterial hypertension (Table 2)^2,3^.

**Table 1:** Total of publications on cardiovascular risk factors between 2001 and 2015 identified by search of MeSH term and revision of the articles’ title and abstract

Year	N	N-Reviewed	% N-Reviewed/N
2001	134	4	2.98%
2002	168	3	1.78%
2003	160	5	3.12%
2004	184	2	1.08%
2005	224	8	3.57%
2006	274	11	4.05%
2007	244	9	3.68%
2008	172	5	2.9%
2009	259	11	4.24%
2010	331	18	5.43%
2011	235	5	2.12%
2012	209	4	1.91%
2013	236	10	4.23%
2014	227	9	3.96%
2015	30	3	10%
Total	3087	107	3.47%

N: Number of articles published; N-Reviewed: Articles selected from the review of abstracts; % N-Reviewed/N: % of articles on cardiovascular risk factors in relation to total number of articles published.

**Table 2:** List of articles selected in 2014 and 2015 and the number of accesses accroding to SciELO database (date of access 06/23/15)

Access	Year	Vol/N/Pags	Article title	Design	Country	Study City
100	2014	V 102, n 4, p 345-354	Dietary interventions and blood pressure In Latin America: systematic review and meta-analysis	SR	Curitiba	Brazil
280	2014	V 102,n 4, p 374-382	Alimentary habits, physical activity, and Framingham global risk score in metabolic syndrome	CSS	Porto Alegre	Brazil
119	2014	V 103,n 21; p 1-31	South American gruidelines for cardiovascular disease prevention and rehabilitation	G	–	Brazil
168	2014	V 103,n 6, p 493-501	Comparison of cardiovascular risk factors in different areas of health care over a 20-year period	CS	Goiânia	Brazil
107	2014	V 102,n 5, p 473-480	Prevalence of cardiovascular risk factors in hemodialysis patients – The CORDIAL study	CSS	Porto Alegre	Brazil
235	2014	V 102,n 6, p 571-578	Blood pressure control In hypertensive patients in the “Hiperdia Program”: a territory-based study	CSS	Porto Alegre	Brazil
151	2014	V 102,n 5, p 420-431	l cardiovascular prevention guideline of the BSC – executive summary	D	–	Brazil

CSS: Cross-sectional study; G: Guidelines; CS: cohort study; SR: Systematic review; BSC: Brazilian Society of Cardiology

In 2005, an ABC editorial discussed the cardiovascular risk factors in Brazil and the perspective of cardiovascular epidemiology in the next 50 years^4^. National data published at that time (one study conducted in São Paulo metropolitan region and the AFIRMAR study)^5,6^, and a study with students about life style and cardiovascular disease^7^ revealed that predictive factors of atherosclerosis in Brazil were not different from those in Europe and North America^8^. In addition, there is a relationship between early mortality caused by cardiovascular disease and social inequality^9^. However, ten years after publication of this editorial, which clarified the definitions of cardiovascular risks in Brazil, most studies about this topic published on ABC have had an observational design. This trend may be found in a review of articles published on ABC in the last 60 years^10^. Therefore, the current scenario is of consolidation and confirmation of traditional risk factors for cardiovascular events, associated with results of mortality rates for ischemic and cerebrovascular disease in different regions of the country.

Therefore, the challenge of cardiovascular epidemiology and of academic publishing in the next years is to promote the development of interventional studies. This approach, in line with primary and secondary prevention measures, may contribute to changes in the epidemiology of cardiovascular risks in Brazil in the coming years. Thus, the role of the leading journal in cardiology in Brazil is to support solid evidence that serve as the basis for practices in our society.
